# mTORC1 Activation Links Chronic Stress to Meso‐Circuit Desynchronization and Accelerated Amyloid Pathology via Autophagic Dysfunction

**DOI:** 10.1002/cns.71174

**Published:** 2026-09-28

**Authors:** Yang Zou, Jun Yang, Yang Li, Guodong Wang, Wen Lu, Xiaoping Chen, Xingxing Ma, Kehan Chen, Peng Liang, Yinghui Wu, Tao Zhuo

**Affiliations:** ^1^ Center for Medical Research The First People's Hospital of Yulin, The Sixth Affiliated Hospital of Guangxi Medical University Yulin Guangxi China; ^2^ The Department of Neurosurgery The First People's Hospital of Yulin, The Sixth Affiliated Hospital of Guangxi Medical University Yulin Guangxi China; ^3^ Department of Pharmacy The First People's Hospital of Yulin, The Sixth Affiliated Hospital of Guangxi Medical University Yulin Guangxi China; ^4^ Department of Oncology The First People's Hospital of Yulin, The Sixth Affiliated Hospital of Guangxi Medical University Yulin Guangxi China; ^5^ Department of Orthopaedic Spine Surgery The First People's Hospital of Yulin, The Sixth Affiliated Hospital of Guangxi Medical University Yulin Guangxi China; ^6^ Center for Respiratory Medicine The First People's Hospital of Yulin, The Sixth Affiliated Hospital of Guangxi Medical University Yulin Guangxi China; ^7^ Department of Pathology The First People's Hospital of Yulin, The Sixth Affiliated Hospital of Guangxi Medical University Yulin Guangxi China; ^8^ Department of Interventional Ultrasound The First People's Hospital of Yulin, The Sixth Affiliated Hospital of Guangxi Medical University Yulin Guangxi China

**Keywords:** Alzheimer's disease, autophagy, depression comorbidity, mTOR signaling, neural network

## Abstract

**Background:**

Although depression is a potent risk factor for Alzheimer's disease (AD), the underlying causal mechanisms remain unclear. This study investigated the molecular and circuit‐level mechanisms linking chronic stress to accelerated AD pathogenesis and evaluated the therapeutic potential of precise mTORC1 targeting.

**Methods:**

A chronic restraint stress (CRS) paradigm was established in 5xFAD mice. Multi‐scale neurofunctional and histopathological alterations were deconstructed using behavioral profiling, wide‐field and two‐photon calcium imaging, immunofluorescence, and bulk RNA‐sequencing. Pharmacological intervention was conducted using the specific mTORC1 inhibitor EN6.

**Results:**

CRS severely impaired cortical slow‐wave oscillations and induced aberrant prefrontal single‐neuron hyperactivity, exacerbating cognitive decline. These network deficits were accompanied by accelerated AD hallmarks, including elevated Aβ deposition, dystrophic neurite aggravation, and reactive gliosis. Mechanistically, transcriptomic profiling and biochemical validation revealed that chronic stress suppresses autophagic pathways via selective hyperactivation of mTORC1 signaling rather than the AMPK pathway. Time‐course analysis showed that mTORC1 activation and autophagy‐related abnormalities preceded overt Aβ accumulation, while pharmacological mTOR activation with MHY1485 further aggravated autophagic impairment and increased Aβ42 levels. Crucially, targeted mTORC1 inhibition with EN6 ameliorated autophagy‐related abnormalities and was associated with reduced BACE1 abundance and CTFβ generation, together with a diminished global Aβ burden. This microenvironmental stabilization attenuated neuroinflammation, realigned neural networks, and rescued both cognitive and emotional deficits.

**Conclusion:**

Chronic stress‐induced mTORC1 hyperactivation is associated with autophagic impairment, contributing to macro‐circuit desynchronization and accelerated amyloid accumulation. Targeting the mTORC1–autophagy axis represents a potential therapeutic approach to mitigate neural network breakdown and neuropathology in stress‐related neurodegenerative conditions.

## Graphical abstract

Chronic stress is associated with mTORC1 hyperactivation and autophagy‐related abnormalities, accompanied by cortical network desynchronization and accelerated amyloid pathology in a 5xFAD model with depression‐like behaviors. Pharmacological mTORC1 inhibition with EN6 ameliorates these molecular, pathological, and network abnormalities and improves recognition memory, supporting the mTORC1–autophagy axis as a potential therapeutic target in stress‐aggravated Alzheimer's disease.

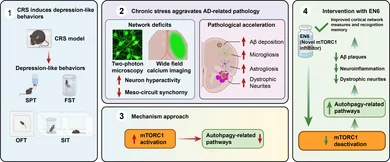

## 1 Introduction

Alzheimer's disease (AD) is a devastating neurodegenerative disorder characterized by progressive cognitive decline, extracellular amyloid‐beta (Aβ) deposition, and robust neuroinflammation secondary to glial activation [1, 2]. While depressive symptoms are frequently observed across the AD spectrum, clinical and epidemiological data regarding its exact role remain conflicting. Prospective cohorts support chronic depressive states as a potent prodromal risk factor that accelerates cognitive deterioration [3, 4, 5, 6, 7], whereas alternative clinical investigations fail to establish a significant causal association [8, 9, 10]. To resolve this ambiguity, we established a chronic restraint stress (CRS)‐induced comorbidity model in 5xFAD mice to systematically deconstruct the multi‐scale impacts of environmental stress on AD molecular pathology and neural network dynamics.

We focused our investigation on the prefrontal cortex (PFC), a critical neural hub integrating advanced cognitive processing and emotional regulation that stands as a primary target for both AD‐ and depression‐associated insults [11, 12, 13]. Homeostatic brain function relies fundamentally on well‐coordinated network rhythms across multiple scales [14]. Macroscopic slow‐wave oscillations (SWO) are indispensable for memory consolidation and metabolic waste clearance, whereas micro‐scale single‐neuron hyperactivity hallmarks early‐stage network destabilization in AD pathology [15, 16, 17]. However, the precise mechanisms by which environmental stress disrupts these multi‐scale network dynamics and selectively induces aberrant cortical firing within the PFC remain completely uncharacterized.

As the cornerstone of the neuronal defense system, autophagy plays a vital protective role in maintaining cerebral homeostasis, clearing proteotoxic aggregates, and suppressing neuroinflammation [18], but is frequently compromised in AD [19]. Within the molecular network modulating autophagy, the mechanistic target of rapamycin (mTOR) and adenosine monophosphate‐activated protein kinase (AMPK) pathways typically exist in a delicate, reciprocal equilibrium [20, 21, 22]. Although the canonical AMPK pathway positively drives autophagic flux in response to metabolic stress [21], the mTOR signaling pathway acts as the core negative regulator of autophagy, functioning as a critical pathogenic node within the cerebral microenvironment shaped by chronic stress [22]. Nevertheless, how these two convergent pathways are pathologically uncoupled under chronic stress, and whether the mTOR or AMPK signaling axes participate in depression‐driven AD aggravation, remains uncharacterized.

Here, using multi‐scale calcium imaging and neuropathological profiling, we demonstrate that chronic restraint stress impairs cortical SWO and induces prefrontal single‐neuron hyperactivity, accompanying accelerated Aβ deposition, reactive gliosis, and dystrophic neurite accumulation. Mechanistically, chronic stress suppresses autophagic degradation via selective hyperactivation of mTORC1 signaling rather than the AMPK pathway. Crucially, pharmacological intervention with the mTORC1 inhibitor EN6 ameliorates autophagy‐related abnormalities and is associated with reduced BACE1 abundance and amyloidogenic APP processing, thereby restoring cortical network synchrony, mitigating plaque‐associated pathologies, and rescuing cognitive and emotional deficits. Together, these findings position the mTORC1–autophagy axis as a promising therapeutic target for addressing stress‐aggravated AD pathology.

## 2 Materials and Methods

### 2.1 Animals and Drug Administration

Both male and female 5xFAD mice (Tg6799 line) and age‐/sex‐matched wild‐type littermates were housed under a standard 12‐h light/dark cycle with ad libitum food and water. All procedures were approved by the Institutional Animal Care and Use Committee of Guangxi Medical University (No. 202605001).

EN6 (CAS 1808714‐73‐9, MCE, HY‐128892, 99.42% purity) was dissolved in 5% DMSO and 95% artificial cerebrospinal fluid (ACSF). A target dose of 2 nmol/mouse was calculated based on the effective in vitro concentration (50 μM) and mouse CSF volume [23]. Accordingly, 1 μL of 2 mM EN6 was unilaterally delivered via intracerebroventricular (i.c.v.) microinjection twice weekly for 4 weeks prior to behavioral testing.

### 2.2 Behavioral Profiling

#### 2.2.1 Chronic Restraint Stress (CRS)

To induce depressive‐like phenotypes, mice were individually restrained in well‐ventilated 50‐mL conical tubes for 2 h daily for 30 consecutive days. Control mice remained undisturbed in their home cages [24].

#### 2.2.2 Sucrose Preference Test (SPT)

Following 24‐h water restriction, mice were presented with two bottles (1% sucrose vs. water) for 3 h. Bottle positions were alternated every 30 min to eliminate spatial preference [24].

#### 2.2.3 Forced Swim Test (FST)

Mice were placed in a clear cylinder filled with water (23°C–25°C). Immobility duration was quantified during the final 4 min of a 6‐min session [25].

#### 2.2.4 Open Field Test (OFT)

Mice were placed at the center of a square arena (50*50*50 cm) for 5 min. Trajectories and center‐zone time were tracked via an overhead digital camera [25].

#### 2.2.5 Social Interaction Test (SIT)

Conducted in a tri‐chambered apparatus. Following a 3‐min habituation, an unfamiliar C57BL/6 stimulus mouse was introduced into a mesh enclosure, and interaction proximity time was quantified over 10 min [24].

#### 2.2.6 Novel Object/Location Recognition Test (NORT/NLRT)

Mice explored two identical objects for 10 min during training. After 24 h, either one object was replaced with a novel one (NORT) or one familiar object was displaced to a new position (NLRT), and the discrimination index was calculated during a 10‐min test phase.

### 2.3 Wide‐Field and Two‐Photon Functional Calcium Imaging

For wide‐field mapping, mice were anesthetized with isoflurane (3% induction, 0.8%–1.0% maintenance). Cortical calcium dynamics were monitored using Cal‐520 AM (AAT Bioquest; 500 nL delivered at 3 nL/s). Epifluorescence imaging was performed on a macroscope with a 2×/0.08 NA lens (Olympus), capturing signals at 30 Hz using an sCMOS camera.

For micro‐scale tracking, 200 nL of AAV2/9‐hSyn‐GCaMP6f‐WPRE‐pA was infused into prefrontal layer 2/3 (+2.8 mm AP, −0.5 mm ML, −0.3 mm DV). Three weeks later, a 3‐mm glass coverslip window was implanted. In vivo two‐photon imaging was executed on a multiphoton microscope with a 12.0‐kHz resonant scanner and a Ti:sapphire laser (920 nm excitation), collecting signals via a 40×/0.8 NA objective at 40 Hz for 130 s.

### 2.4 Intracerebroventricular (i.c.v.) Cannulation

A stainless‐steel guide cannula was stereotaxically lowered into the right lateral ventricle (coordinates relative to bregma: AP = 0.0 mm, ML = 1.0 mm, DV = −2.0 mm from dura) under isoflurane anesthesia and anchored to the skull using dental cement.

### 2.5 Histopathology and Congo Red Staining

Mice were transcardially perfused with ice‐cold saline. Contralateral hemispheres were fixed in 4% PFA and cryoprotected in 30% sucrose. Coronal sections (50 μm) cut via a cryostat (Thermo Scientific CryoStar NX50) were incubated in 0.02% Congo red for 15 min, mounted with DAPI‐enriched antifade medium, and mapped via a Zeiss LSM910 confocal microscope.

### 2.6 Tissue Biochemical Extraction and ELISA

Cryopreserved brain tissues were pulverized in liquid nitrogen. Tissues were homogenized in TBS and centrifuged to collect the aqueous‐soluble fraction (supernatant). The pellet was reconstituted in SDS lysis buffer and centrifuged identically to harvest the detergent‐soluble fraction. The remaining debris was extracted with 100% formic acid (FA), neutralized, and centrifuged to isolate the FA‐soluble fraction. Aβ levels were quantified via commercial ELISA kits (Elabscience, E‐UNEL‐H0256 and E‐EL‐H0543) and normalized to total protein concentration.

### 2.7 Immunofluorescence Staining

Coronal sections underwent antigen retrieval in 10 mM sodium citrate buffer at 95°C for 10 min. Sections were blocked and permeabilized with 10% normal donkey serum and 1% Triton X‐100 for 1 h, incubated with primary antibodies overnight at 4°C, and exposed to species‐specific fluorophore‐conjugated secondary antibodies for 2 h at room temperature prior to Zeiss LSM910 confocal imaging (antibody information can be found in Table S1).

### 2.8 Bulk RNA‐Sequencing and Bioinformatic Analysis

PFC tissues (+2.0 to +3.0 mm relative to bregma) were micro‐dissected under a stereomicroscope, snap‐frozen, and shipped to LC‐Bio Technology (Hangzhou, China) for mRNA library construction and sequencing. Bioinformatic visualization and enrichment analysis workflows were performed using the OmicStudio platform.

### 2.9 Western Blot

Prefrontal cortical tissues were homogenized in ice‐cold RIPA buffer containing protease and phosphatase inhibitors, incubated on ice, and centrifuged. Proteins were denatured in loading buffer at 95°C for 5 min, separated via 10% SDS‐PAGE, and transferred onto PVDF membranes. Blots were blocked, incubated with primary antibodies overnight at 4°C, and exposed to HRP‐conjugated secondary antibodies for 1 h (Antibody information can be found in Table S1). Protein bands were visualized via enhanced chemiluminescence (Beyotime) on an Odyssey XF imager and quantified via ImageJ (Fiji).

### 2.10 Statistical Analysis

Analyses were performed using GraphPad Prism (version 9.4.0). Data are presented as mean ± SEM. The mouse was considered the biological experimental unit for inferential statistical analyses. For histological and imaging datasets in which multiple neurons, plaques, glial cells, dystrophic neurites, or fields of view were obtained from the same animal, individual observations were first summarized within each mouse, and the resulting animal‐level values were used for statistical comparisons. Pooled individual‐object data were used only for visualization of distributions where indicated and were not treated as independent biological replicates.

Normality of animal‐level data was assessed using the Shapiro–Wilk test. For comparisons between two independent groups, an unpaired two‐tailed Student's *t*‐test was used for normally distributed data, whereas the Mann–Whitney test was applied to non‐normally distributed data. Comparisons among multiple independent groups were performed using one‐way ANOVA followed by Tukey's multiple‐comparisons test. For datasets containing repeated measurements from the same animal across cortical regions or anatomical distances, two‐way repeated‐measures ANOVA followed by Bonferroni's multiple‐comparisons test was used. The numbers of animals and individual observations are specified separately in the corresponding figure legends. Statistical significance was defined as *p* < 0.05.

## 3 Results

### 3.1 Chronic Stress Disrupts Cortical Slow‐Wave Oscillations and Induces Prefrontal Neuronal Hyperactivity in 5xFAD Mice

To investigate how comorbid depression affects AD progression, 2‐month‐old 5xFAD mice underwent a 4‐week CRS paradigm (Figure 1a). Behavioral profiling across four groups (WT, WT + CRS, 5xFAD, 5xFAD + CRS) showed that CRS induced robust emotional deficits, evidenced by decreased sucrose preference (Figure 1b), prolonged immobility in the FST (Figure 1c), reduced central zone time in the OFT (Figure 1d), and a decreased social interaction ratio in the SIT (Figure 1e). Cognitive assessments via NORT and NLRT revealed that comorbid 5xFAD + CRS mice exhibited a catastrophic drop in the discrimination index (Figure 1f,g), demonstrating a synergistic aggravation of recognition and spatial memory consolidation.

**FIGURE 1:**
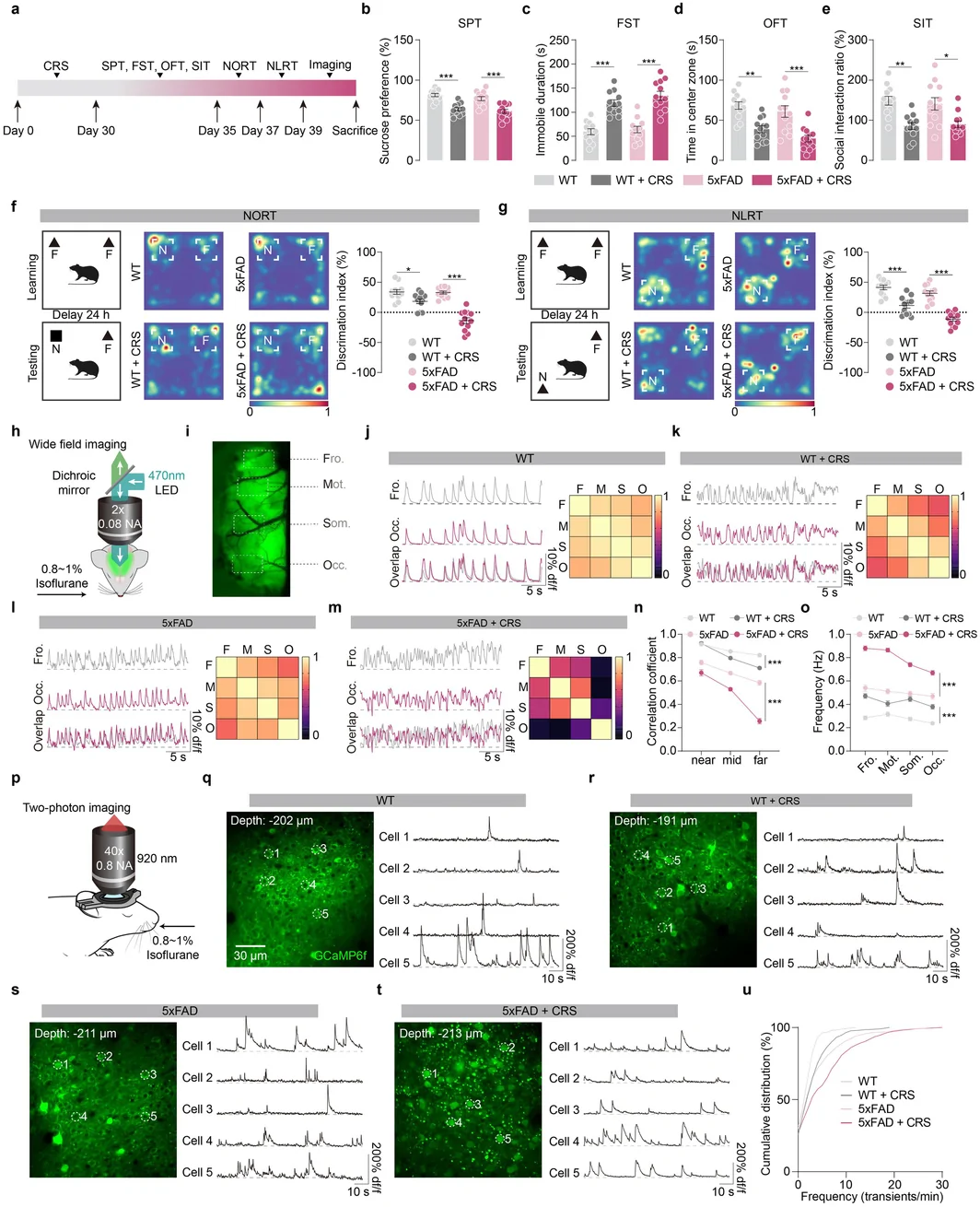
Comorbid depression disrupts cortical slow‐wave oscillations and induces prefrontal neuronal hyperactivity in 5xFAD mice. (a) Experimental timeline. Mice were subjected to chronic restraint stress (CRS) for 30 days, followed by a battery of behavioral tests and in vivo imaging, leading to sacrifice. (b) Statistical plot shows the percentage of sucrose preference in the sucrose preference test (SPT) across the four experimental groups (*n* = 12 mice per group, one‐way ANOVA). (c) Statistical plot shows the immobility duration in the forced swim test (FST) across the experimental groups (*n* = 12 mice per group, one‐way ANOVA). (d) Statistical plot shows the time spent in the center zone during the open field test (OFT) across different cohorts (*n* = 12 mice per group, one‐way ANOVA). (e) Statistical plot shows the social interaction ratio during the social interaction test (SIT) across the experimental cohorts (*n* = 12 mice per group, one‐way ANOVA). (f) Left, schematic of the novel object recognition test (NORT) experimental paradigm; mid, representative occupancy heatmaps illustrating object exploration trajectory during the testing phase; right, statistical plot showing the discrimination index during the testing phase of the NORT (*n* = 12 mice per group, one‐way ANOVA). (g) Left, schematic of novel location recognition test (NLRT) experimental paradigm; mid, representative occupancy heatmaps illustrating location exploration trajectory during the testing phase; right, statistical plot showing the discrimination index during the testing phase of the NLRT (*n* = 12 mice per group, one‐way ANOVA). (h) Schematic diagram depicting the in vivo wide‐field calcium imaging configuration, equipped with a high‐speed sCMOS camera and 470 nm LED illumination, in isoflurane‐anesthetized mice. (i) Representative wide‐field calcium image showing the field of view over multiple cortical regions, including Frontal (Fro.), Motor (Mot.), Somatosensory (Som.), and Occipital (Occ.) cortices. (j–m) Representative calcium fluorescence traces (left) and corresponding inter‐regional correlation matrices (right) illustrate the spatiotemporal distribution of SWO across four distinct cortical areas—frontal, motor, somatosensory, and occipital cortices—in WT, WT + CRS, 5xFAD, and 5xFAD + CRS mice, highlighting the stress‐induced disruption of cortical synchronization. (n) Spatial correlation profiles of cortical calcium signals plotted as a function of anatomical distance from the reference region (categorized into near (Fro‐Mot, Mot‐Som, and Som‐Occ), mid (Fro‐Som and Mot‐Occ), and far (Fro‐Occ) bins) (*n* = 12 mice per group, two‐way ANOVA). (o) Frequencies (Hz) of SWO quantified across distinct cortical regions, including the frontal, motor, somatosensory, and occipital cortices (*n* = 12 mice per group, two‐way ANOVA). (p) Two‐photon calcium imaging setup. Schematic illustration of the in vivo two‐photon microscope. (q–t) Representative prefrontal cortex (PFC) calcium imaging data. Panels show prefrontal cortex images (left) with specified imaging depths, showing individual neuron identifications (labeled cells 1–5). Associated calcium fluorescence traces (right) from the five identified cells are plotted for WT, WT + CRS, 5xFAD, and 5xFAD + CRS mice. (u) Cumulative distribution of PFC neuron calcium transient frequencies (transients/min). Error bars represent mean ± SEM. *p < 0.05, **p < 0.01, ***p < 0.001, ns = no significance.

Given that cognitive processing depends on large‐scale network dynamics [16, 26, 27], we performed wide‐field functional calcium imaging to map macro‐circuit SWO across frontal, motor, somatosensory, and occipital cortices (Figure 1h,i). Healthy WT mice displayed highly synchronized, rhythmic SWO with correlation coefficients near unity (Figure 1j). In contrast, chronic stress or AD pathology progressively decoupled these macro‐circuits (Figure 1k,l), culminating in a profound breakdown of global network synchrony in 5xFAD + CRS mice, manifested by fragmented calcium traces, collapsed correlation matrices (Figure 1m), and reduced correlation coefficients across near, mid, and far distances (Figure 1n). Concurrently, the calcium frequency significantly increased across all cortical regions in comorbid mice (Figure 1o), indicating macro‐circuit desynchronization.

To unravel the underlying micro‐circuit mechanisms, we deployed in vivo two‐photon calcium imaging to track layer II/III PFC neurons (Figure 1p). Firing profiles of single neurons were stratified into silent, normal, or hyperactive subpopulations (Figure 1q–t; Figure S1a–d). In WT mice, the network was dominated by normal‐firing neurons (66.2%) with minimal hyperactivity (3.7%) (Figure S1a). Stress or AD pathology independently elevated hyperactive neuron proportions to 18.3% and 24.2%, respectively (Figure S1b,c). Strikingly, while the proportion of silent neurons remained invariant across all groups (Figure S1e), comorbid 5xFAD + CRS pathology synergistically expanded the hyperactive pool to 40.3% while compressing the normal reserve to 32.9% (Figure S1d,f,g), inducing a massive rightward shift in the cumulative frequency distribution (Figure 1u) and a stepwise increase in the mean firing rate (Figure S1h).

### 3.2 Depression‐Like Phenotypes Accelerate Amyloid Accumulation, Plaque‐Associated Axonal Dystrophy, and Reactive Gliosis

To establish the histopathological substrates driving network collapse, we examined cortical neuropathology in 5xFAD mice. Congo red staining revealed that 5xFAD + CRS mice exhibited a striking elevation in dense‐core plaque burden compared to 5xFAD controls (Figure 2a), with enlarged individual plaque sizes (Figure 2b) and increased plaque density (Figure 2c). Sequential protein extraction and ELISA quantification showed that chronic stress significantly elevated the concentrations of both Aβ40 and highly toxic Aβ42 species across soluble (TBS), detergent‐soluble (SDS), and insoluble (FA) homogenates (Figure 2d,e), biochemically confirming that a depressive state accelerates multi‐fraction amyloid accumulation.

**FIGURE 2:**
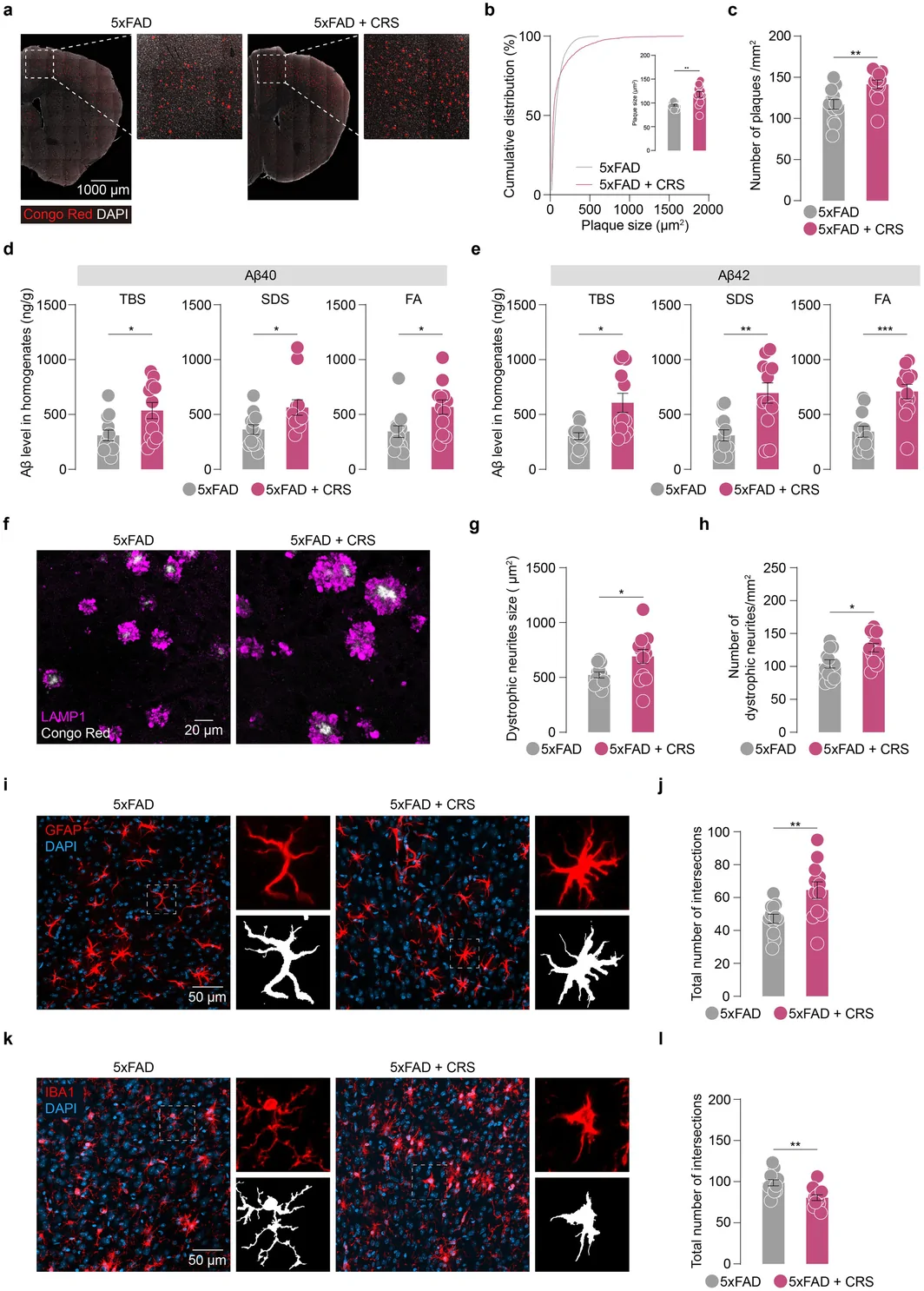
CRS‐induced depression exacerbates Aβ deposition, axonal dystrophy, and neuroinflammation in 5xFAD mice. (a) Representative panoramic and high‐magnification confocal images of brain sections from PFC of 5xFAD and 5xFAD + CRS mice, stained with Congo red (red, amyloid plaques) and DAPI (gray, nuclei). (b) Cumulative frequency distribution of amyloid plaque size. Inset histogram quantifies the plaque size (*n* = 12 mice per group, 61–235 plaques analyzed and averaged per mouse, Mann–Whitney test). (c) Quantification of amyloid plaque density in PFC, expressed as the number of plaques per square millimeter (*n* = 12 mice per group, 3–5 fields of view analyzed and averaged per mouse, Mann–Whitney test). (d) ELISA quantification of Aβ40 concentrations in TBS‐, SDS‐, and FA‐soluble fractions, sequentially extracted from cortical homogenates (*n* = 12 mice per group, Mann–Whitney test). (e) ELISA quantification of Aβ42 concentrations in TBS‐, SDS‐, and FA‐soluble fractions, sequentially extracted from cortical homogenates (*n* = 12 mice per group, Mann–Whitney test). (f) Representative high‐resolution confocal micrographs demonstrating dystrophic neurites surrounding the periphery of amyloid plaques, co‐stained for LAMP1 (dystrophic neurites marker) and plaque cores (Congo red, white). (g) Statistical analysis of plaque‐associated dystrophic neurites size (*n* = 12 mice per group, 4–8 dystrophic neurites analyzed and averaged per mouse; Mann–Whitney test). (h) Statistical analysis of plaque‐associated dystrophic neurites density (*n* = 12 mice per group, 2–3 fields of view analyzed and averaged per mouse, Mann–Whitney test). (i) Representative immunostaining images of astrogliosis in the prefrontal cortex, labeled with GFAP (red) and DAPI (blue). Right panels show magnified single‐astrocyte morphology (top) and the corresponding skeletonized reconstruction used for morphometric profiling (bottom). (j) Quantitative morphometric evaluation of astrocyte complexity, indicated by the total number of intersections derived from Sholl analysis (*n* = 12 mice per group, 2–4 astrocytes analyzed and averaged per mouse, Mann–Whitney test). (k) Representative immunostaining images of microglia in the prefrontal cortex, labeled with IBA1 (red) and DAPI (blue). Right panels display magnified single‐microglia morphology (top) and the corresponding skeletonized reconstruction used for morphometric profiling (bottom). (l) Quantitative analysis of microglial ramification, showing the total number of intersections from Sholl analysis (*n* = 12 mice per group, 3–4 microglia analyzed and averaged per mouse, Mann–Whitney test). Each dot represents an individual animal. Error bars represent mean ± SEM. *p < 0.05, **p < 0.01, ***p < 0.001, ns = no significance.

We next analyzed plaque‐associated axonal dystrophy by co‐labeling cortical sections with Congo red and LAMP1 (Figure 2f). Morphometric profiling revealed that the size of dystrophic neurites haloing dense plaque cores expanded significantly in the 5xFAD + CRS group, coupled with a robust increase in their numerical density (Figure 2g,h), demonstrating that stress‐accelerated amyloid deposition exacerbates peri‐plaque axonal structural damage.

To profile reactive neuroinflammation, we examined cortical astrocytes and microglia. GFAP immunostaining mapped prominent reactive astrogliosis (Figure 2i). Sholl analysis of three‐dimensional skeletonized reconstructions showed a significant increase in astrocytic process intersections (Figure 2j). Single‐cell profiling further revealed a significant expansion of astrocytic soma area and total process length, though spatial density increased without statistical significance (Figure S2a–c). In stark contrast, IBA1 staining mapped a completely divergent microglial program (Figure 2k). Sholl analysis unmasked a severe reduction in microglial intersections (Figure 2l), indicating a shift toward a de‐ramified phenotype. Extended stereological assessments revealed massive microglial proliferation (increased cell density), characterized by somatic swelling but a significant retraction of total process length (Figure S2d–f).

### 3.3 Chronic Stress Suppresses Cortical Autophagy Through Aberrant mTOR Activation Independently of AMPK Signaling

To elucidate the molecular mechanisms by which chronic stress accelerates AD pathology, we performed bulk RNA‐seq on PFC tissues from 5xFAD and 5xFAD + CRS mice (Figure 3a). Transcriptomic analysis identified a distinct profile of differentially expressed genes (DEGs) between the two cohorts (Figure 3a). KEGG and GO enrichment analyses revealed that these DEGs were prominently enriched in cellular and immune processes, including phagosome, apoptosis, lysosome, signal transduction, and innate immune response (Figure 3b,c). Crucially, Gene Set Enrichment Analysis (GSEA) demonstrated that chronic stress significantly enriched gene sets associated with Alzheimer's disease, immune response, and amyloid‐beta binding (Figure 3d–f), while unmasking a profound enrichment in the negative regulation of autophagy gene set (Figure 3g). The depressive phenotype is associated with autophagy, thereby exacerbating the Aβ pathology in the cerebral cortex.

**FIGURE 3:**
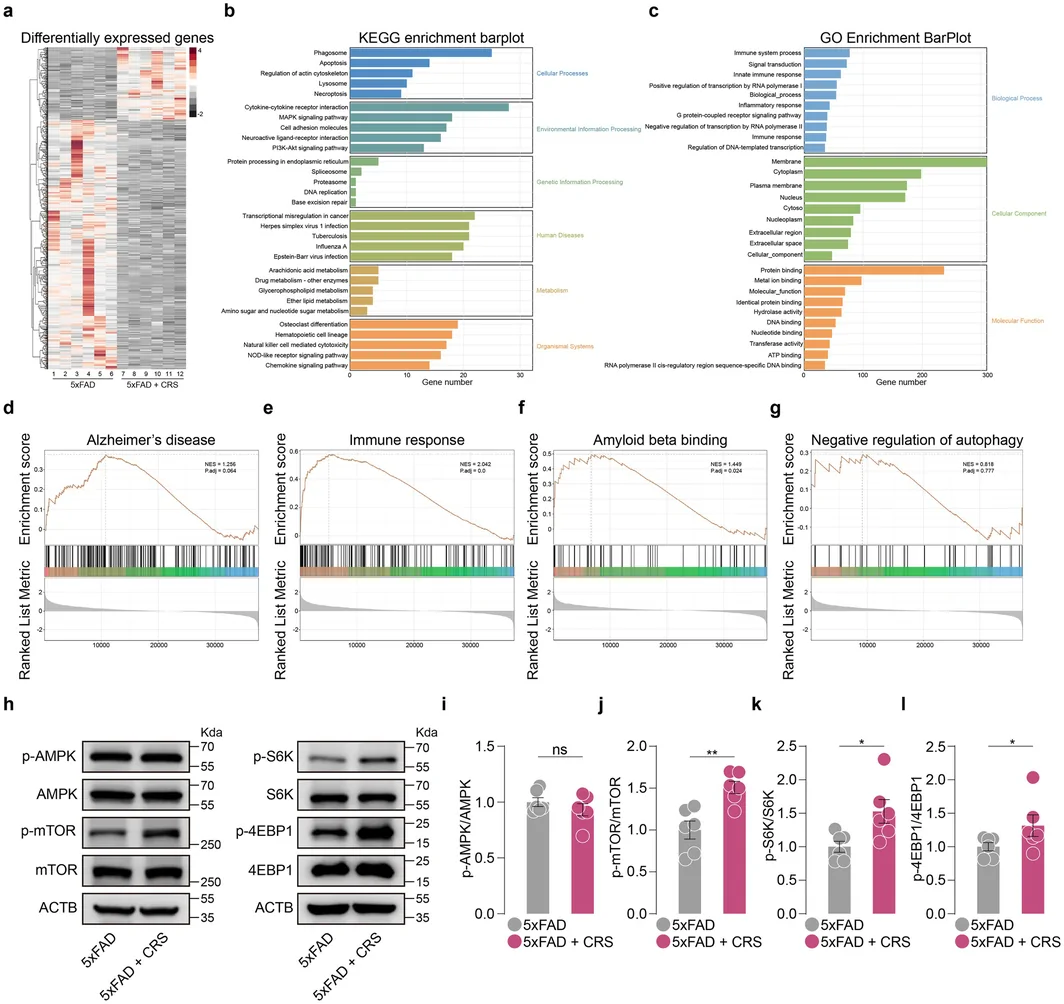
CRS‐induced depression suppresses cortical autophagy via mTOR activation independently of AMPK signaling in the AD brain. (a) Hierarchical clustering heatmap displaying differentially expressed genes (DEGs) in the prefrontal cortex between 5xFAD and 5xFAD + CRS mice (*n* = 6 mice per group). The color scale indicates normalized expression levels (*z*‐score), ranging from down‐regulation (gray) to up‐regulation (red). (b) KEGG pathway enrichment bar plot of the DEGs, categorized by functional themes including cellular processes, environmental information processing, genetic information processing, human diseases, metabolism, and organismal systems. (c) Gene Ontology (GO) enrichment bar plot illustrating the top enriched terms classified into three major categories: biological process, cellular component, and molecular function. (d–g) Gene Set Enrichment Analysis (GSEA) plots evaluating transcripts associated with key pathological and functional gene sets. (h) Representative western blot bands showing the protein and phosphorylation levels of p‐AMPK, AMPK, p‐mTOR, mTOR, p‐S6K, S6K, p‐4EBP1, and 4EBP1 in cortical homogenates from 5xFAD and 5xFAD + CRS mice. (i–l) Quantification of the ratio of phosphorylated protein to total protein, including: P‐AMPK/AMPK, p‐mTOR/mTOR, p‐S6K/S6K, and p‐4EBP1/4EBP1 (*n* = 6 mice per group, Mann–Whitney test). Each dot represents an individual animal. Error bars represent mean ± SEM. *p < 0.05, **p < 0.01, ***p < 0.001, ns = no significance.

To validate the upstream enzymatic cascade, we examined the master autophagy‐regulating hubs—AMPK and mTOR [21, 22]—via Western blotting (Figure 3h). Immunoblotting demonstrated that the p‐AMPK (Thr172)/AMPK ratio remained unaltered between 5xFAD and 5xFAD + CRS cohorts (Figure 3i), eliminating AMPK inactivation as a driver of the autophagic defect. In stark contrast, the mTOR signaling cascade exhibited robust hyperactivation; chronic stress induced a significant elevation in the p‐mTOR (Ser2481)/mTOR ratio (Figure 3h,j). To confirm downstream signaling competency, we examined canonical substrates of mTORC1: p70S6 Kinase (S6K) and 4E‐binding protein 1 (4EBP1) (Figure 3h). Quantitative profiling revealed a significant increase in the phosphorylation ratios of both p‐S6K/S6K and p‐4EBP1/4EBP1 in comorbid 5xFAD + CRS brains (Figure 3k,l).

### 3.4 mTORC1 Activation and Autophagic Suppression Precede Aβ Accumulation During CRS Progression Independently of mTORC2

To determine the temporal relationship among stress‐induced mTOR signaling, autophagy‐related abnormalities, and amyloid pathology, we mapped the longitudinal time‐course kinetics in 5xFAD mice subjected to CRS across 0, 7, 14, and 30 days (Figure S3a).

Western blot analysis of cortical tissues revealed a progressive, time‐dependent increase in total mTOR phosphorylation (p‐mTOR/mTOR), which exhibited an upward trend at Day 7, increased further at Day 14, and peaked at Day 30 (Figure S3b,e). To delineate which specific mTOR complex was engaged, we examined downstream targets of mTORC1 (p‐S6K) and mTORC2 (p‐AKT). Notably, p‐S6K/S6K levels mirrored the kinetics of p‐mTOR, showing an increasing trend starting at Day 7 and escalating further by Day 30 (Figure S3c,f). In stark contrast, p‐AKT/AKT levels remained unaltered throughout the entire 30‐day CRS course (Figure S3d,g), demonstrating that chronic environmental stress mainly drives mTORC1, but not mTORC2, hyperactivation in the cortical microenvironment.

We next examined temporal changes in autophagy‐related markers in relation to amyloid biogenesis. Paralleling the onset of mTORC1 overactivation, cortical autophagy‐associated molecular changes emerged early during CRS progression. Specifically, levels of the autophagic substrate p62/ACTB increased as early as Day 7 and accumulated extensively by Day 30 (Figure S3i,k), while the autophagosome‐associated marker LC3B/ACTB displayed a biphasic trajectory characterized by an initial increase followed by a subsequent decline (Figure S3i,j). Crucially, ELISA quantification revealed that TBS‐soluble Aβ42 levels showed an increasing trend at Day 14 and underwent a significant surge at Day 30 (Figure S3h).

Together, these chronological kinetics demonstrate that mTORC1 hyperactivation and alterations in autophagy‐related markers precede macroscopic Aβ42 accumulation, supporting an early association between mTORC1 activation, autophagic impairment, and subsequent amyloid pathology.

### 3.5 Pharmacological Treatment With the mTOR Activator MHY1485 Is Associated With Altered Autophagy‐Related Markers and Increased Aβ42 Levels

Having established that mTORC1 hyperactivation precedes amyloid accumulation during stress progression, we next examined the effects of pharmacological treatment with MHY1485 in 5xFAD mice. To provide pharmacological gain‐of‐function support, 5xFAD mice were treated with MHY1485, an mTOR activator, as previously described [28] (Figure S4a).

Western blot analysis showed that MHY1485 treatment was associated with marked alterations in cortical autophagy‐related markers. Compared with vehicle‐treated controls, MHY1485 administration decreased LC3B/ACTB (Figure S4b,c) and increased p62/ACTB (Figure S4b,d), consistent with impaired autophagic activity.

ELISA quantification further showed that these changes in autophagy‐related markers were accompanied by a marked increase in TBS‐soluble Aβ42 levels in cortical tissue following MHY1485 treatment (Figure S4e).

Collectively, these pharmacological findings show that MHY1485 treatment is associated with alterations in autophagy‐related markers and increased soluble Aβ42 levels, providing supportive evidence for a relationship between enhanced mTOR signaling, altered autophagic activity, and amyloid accumulation in the AD brain.

### 3.6 Pharmacological Inhibition of mTORC1 by EN6 Ameliorates Depressive Behaviors and Rescues Multi‐Scale Neurofunctional Deficits

To determine whether targeting the aberrant mTOR axis could rescue neurofunctional deficits, 5xFAD + CRS mice received unilateral i.c.v. microinjections of EN6 (1 μL of 2 mM solution, twice weekly for 4 weeks; Figure 4a), a small‐molecule v‐ATPase covalent ligand that inactivates mTORC1 to promote autophagy without triggering compensatory Akt activation [23].

**FIGURE 4:**
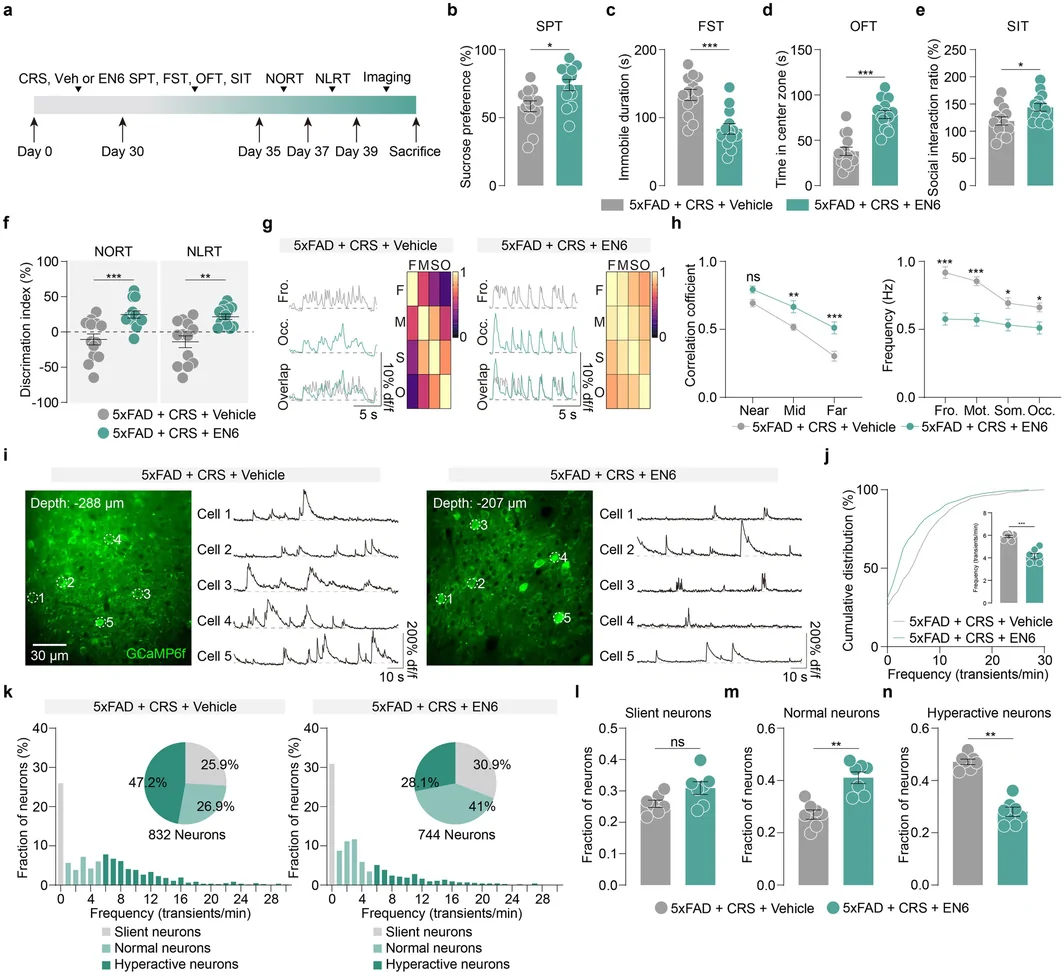
mTOR inhibition by EN6 ameliorates depressive‐like behaviors and reverses multi‐scale neurofunctional impairments in a mouse model of depression‐AD comorbidity. (a) Schematic timeline of the experimental design. Comorbid 5xFAD mice subjected to CRS were randomized to receive either vehicle or the mTOR inhibitor EN6, followed by sequential assessment via behavioral assays and in vivo calcium imaging prior to sacrifice. (b) Statistical plot shows the percentage of sucrose preference in the SPT across the experimental groups (*n* = 13 mice per group, one‐way ANOVA). (c) Statistical plot shows the immobility duration in the FST across the experimental groups (*n* = 13 mice per group, one‐way ANOVA). (d) Statistical plot shows the time spent exploring the central zone in the OFT across the experimental groups (*n* = 13 mice per group, one‐way ANOVA). (e) Statistical plot shows the social interaction ratio in the SIT across the experimental groups (*n* = 13 mice per group, one‐way ANOVA). (f) Statistical analysis of cognitive memory recovery, showing the discrimination index during the testing phases of the NORT and NLRT (*n* = 13 mice per group, one‐way ANOVA). (g) Representative calcium signal traces (left) and corresponding inter‐regional functional connectivity matrices (right) illustrating cortical SWO across frontal (F), motor (M), somatosensory (S), and occipital (O) areas in vehicle‐ and EN6‐treated comorbid 5xFAD mice. (h) Statistical quantification of SWO rescue effects. Left: Spatial correlation coefficients plotted as a function of anatomical distance from the reference region (*n* = 13 mice per group, two‐way ANOVA). Right: Mean SWO frequency profiles across the specified cortical regions (*n* = 13 mice per group, two‐way ANOVA). (i) In vivo two‐photon calcium imaging of PFC neurons. Panels show representative micrographs identifying single neurons (labeled cells 1–5, left) and their corresponding relative calcium changes (ΔF/F) plotted over time (right). (j) Cumulative distribution of single‐neuron transient frequency. Inset graph demonstrates the statistical reduction of calcium transient frequency under EN6 administration (*n* = 7 mice per group, Mann–Whitney test). (k) Distribution histograms showing the fraction of total recorded neurons. Inset pie charts classify cells based on firing rates into three functional subpopulations: Silent neurons (gray), normal neurons (light green), and hyperactive neurons (dark green). (l–n) Statistical quantification comparing the fraction of neurons per animal across groups for silent, normal, and hyperactive neural subpopulations, highlighting that EN6 selectively dampens pathological PFC hyperexcitability (*n* = 7 mice per group, Mann–Whitney test). Each dot represents an individual animal. Error bars represent mean ± SEM. *p < 0.05, **p < 0.01, ***p < 0.001, ns = no significance.

Western blot analysis confirmed that central administration of EN6 effectively dampens cortical mTOR signaling, leading to a marked decrease in p‐mTOR/mTOR expression in 5xFAD + CRS mice compared with vehicle‐treated controls (Figure S5a,b). Importantly, EN6 treatment dampened p‐S6K phosphorylation without inducing compensatory phosphorylation of p‐AKT (Figure S5c–f), confirming its mTORC1‐specific inhibitory action [23].

In behavioral assays targeting emotional reactivity, EN6‐treated mice displayed an increased sucrose preference in the SPT (Figure 4b), a significantly truncated immobility duration in the FST (Figure 4c), and increased central zone time in the OFT (Figure 4d), accompanied by an enhanced social interaction ratio in the SIT (Figure 4e). Furthermore, EN6 robustly reversed cognitive deficits, yielding a significant recovery of the discrimination index in both the NORT and NLRT (Figure 4f).

To evaluate macro‐circuit coordination, we deployed wide‐field calcium imaging to profile cortical SWO (Figure 4g). While vehicle‐treated comorbid mice exhibited severely disrupted network synchrony, EN6 treatment realigned large‐scale networks into highly synchronized, rhythmic slow‐wave oscillatory sweeps (Figure 4g). Quantitative analysis confirmed that EN6 significantly elevated correlation coefficients across mid‐ and far‐range anatomical distances, while normalizing dominant oscillation frequencies across the frontal, motor, somatosensory, and occipital cortices (Figure 4h).

To uncover the cellular foundations, we utilized in vivo two‐photon imaging to monitor layer II/III PFC neurons (Figure 4i). EN6 suppressed chaotic, high‐frequency hyperactive firing, driving a distinct leftward shift in the cumulative frequency distribution and a significant reduction in the mean firing rate (Figure 4i,j). Subpopulation stratification across 832 vehicle‐treated and 744 EN6‐treated neurons revealed that while the silent fraction remained stable (Figure 4k,l), EN6 precisely remodeled active states, robustly expanding the homeostatic normal‐firing pool from 26.9% to 41.0% (Figure 4k,m) while reciprocally draining the pathological hyperactive population from 47.2% down to 28.1% (Figure 4k,n).

### 3.7 EN6 Attenuates Aβ Burden by Shifting the APP Processing Pathway

To identify the histopathological and biochemical drivers of EN6 efficacy, we mapped cortical amyloid deposition via Congo red staining (Figure 5a). Confocal visualization revealed a striking reduction in dense‐core amyloid plaque burden in EN6‐treated 5xFAD + CRS mice compared to vehicle controls (Figure 5a). Morphometric modeling illustrated a prominent leftward shift in individual plaque sizes, a significant contraction in mean plaque diameter (Figure 5b), and a substantial reduction in global plaque density (Figure 5c). Serial tissue fractionations and ELISA profiling confirmed that EN6 induced a profound reduction in the concentrations of both Aβ40 and Aβ42 across soluble (TBS), membrane‐associated (SDS), and insoluble (FA) homogenates (Figure 5d,e).

**FIGURE 5:**
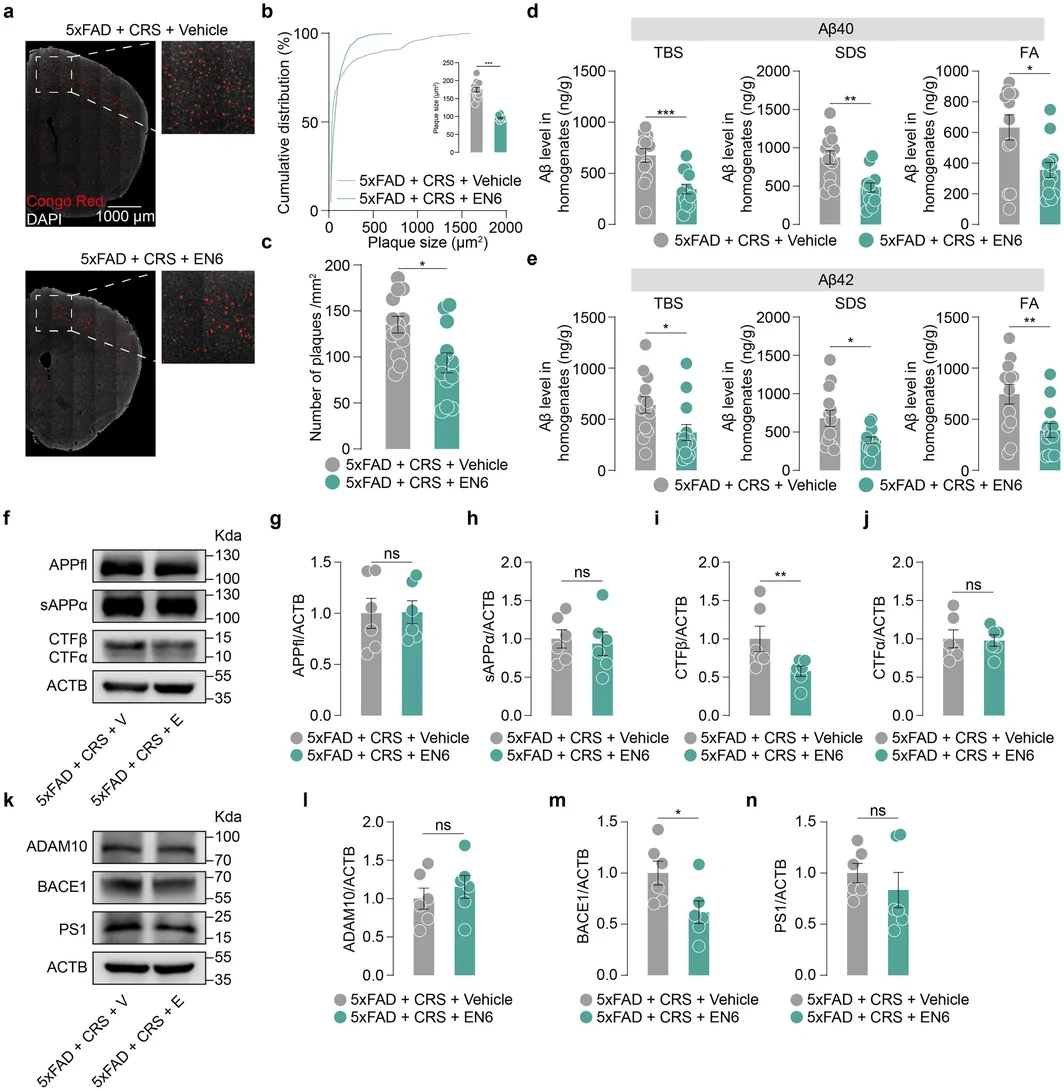
EN6 attenuates amyloid deposition by shifting the APP processing pathway in depression‐AD comorbidity. (a) Representative panoramic and high‐magnification confocal micrographs of coronal brain sections containing the PFC from 5xFAD + CRS + Vehicle and 5xFAD + CRS + EN6 mice, co‐stained with Congo red (red, amyloid plaques) and DAPI (gray, nuclei). (b) Statistical assessment of PFC amyloid plaque burden. Cumulative distribution of amyloid plaque size. Inset plot quantifies the plaque size (*n* = 13 mice per group, 93–209 plaques analyzed and averaged per mouse, Mann–Whitney test). (c) Statistical assessment of PFC plaque density (*n* = 13 mice per group, 3–5 fields of view analyzed and averaged per mouse, Mann–Whitney test). (d) ELISA quantification of Aβ40. Peptide concentrations (ng/g) in TBS‐, SDS‐, and FA‐soluble fractions sequentially extracted from cortical homogenates (*n* = 13 mice per group, Mann–Whitney test). (e) ELISA quantification of Aβ42. Peptide concentrations (ng/g) in TBS‐, SDS‐, and FA‐soluble fractions sequentially extracted from cortical homogenates (*n* = 13 mice per group, Mann–Whitney test). (f) Representative blot bands showing the protein expression levels of full‐length APP (APPfl), soluble APPα (sAPPα), and C‐terminal fragments CTFβ and CTFα in cortical tissue from the indicated groups. (g–j) Quantification of protein abundance normalized to ACTB, including: APPfl/ACTB, sAPPα/ACTB, CTFβ/ACTB, and CTFα/ACTB (*n* = 6 mice per group, Mann–Whitney test). (k) Representative blot bands of APP‐cleaving secretases, including ADAM10 (α‐secretase), BACE1 (β‐secretase), and PS1 (γ‐secretase catalytic subunit) in cortical homogenates. (l–n) Densitometric analysis of secretase expression normalized to ACTB: ADAM10/ACTB, BACE1/ACTB, and PS1/ACTB (*n* = 6 mice per group, Mann–Whitney test). Each dot represents an individual animal. Error bars represent mean ± SEM. *p < 0.05, **p < 0.01, ***p < 0.001, ns = no significance.

Concurrently, EN6 treatment was associated with changes in autophagy‐related markers consistent with improved autophagic activity in the comorbid brain. Following EN6 treatment, levels of the autophagosome‐associated marker LC3B/ACTB were significantly restored (Figure S5g,h), whereas the accumulated autophagic substrate p62/ACTB underwent a profound reduction (Figure S5g,i), consistent with amelioration of autophagy‐related abnormalities.

To examine the contribution of autophagic‐lysosomal degradation to the anti‐amyloidogenic effect of EN6, we performed a pathway‐dependency experiment by co‐administering chloroquine (CQ), a lysosomal degradation inhibitor (Figure S5j). ELISA quantification revealed that pharmacological inhibition of autophagy by CQ significantly abated the anti‐amyloidogenic effect of EN6, resulting in a marked rebound of TBS‐soluble Aβ42 levels in 5xFAD + CRS cortical homogenates (Figure S5k).

To resolve the precise enzymatic mechanisms, we deconstructed the APP cleavage cascade via Western blotting (Figure 5f). Expression of APPfl remained statistically unaltered between vehicle‐ and EN6‐treated groups (Figure 5f,g). In the non‐amyloidogenic pathway, neither sAPPα (Figure 5f,h) nor membrane‐bound CTFα (Figure 5f,j) exhibited significant expression changes. However, CTFβ abundance was significantly reduced following EN6 treatment (Figure 5f,i), consistent with reduced amyloidogenic APP processing.

To identify the specific secretases directing this shift, we evaluated ADAM10, BACE1, and PS1 expression (Figure 5k). Consistent with sAPPα and CTFα data, ADAM10 expression remained unaltered (Figure 5l). BACE1 abundance was significantly reduced following EN6 treatment (Figure 5m), whereas ADAM10 and PS1 protein levels remained unchanged (Figure 5l,n).

### 3.8 EN6 Mitigates Peri‐Plaque Axonal Dystrophy and Alleviates Reactive Neuroinflammation in Comorbid Mice

To determine whether EN6‐mediated amyloid reduction restores microenvironmental stability, we characterized peri‐plaque axonal damage in the PFC via Congo red and LAMP1 co‐labeling (Figure 6a). Confocal morphometric profiling revealed that EN6 significantly suppressed the expansion of lysosome‐enlarged dystrophic neurites surrounding dense plaque cores (Figure 6a), yielding significant reductions in both individual neurite size (Figure 6b) and overall numerical density (Figure 6c).

**FIGURE 6:**
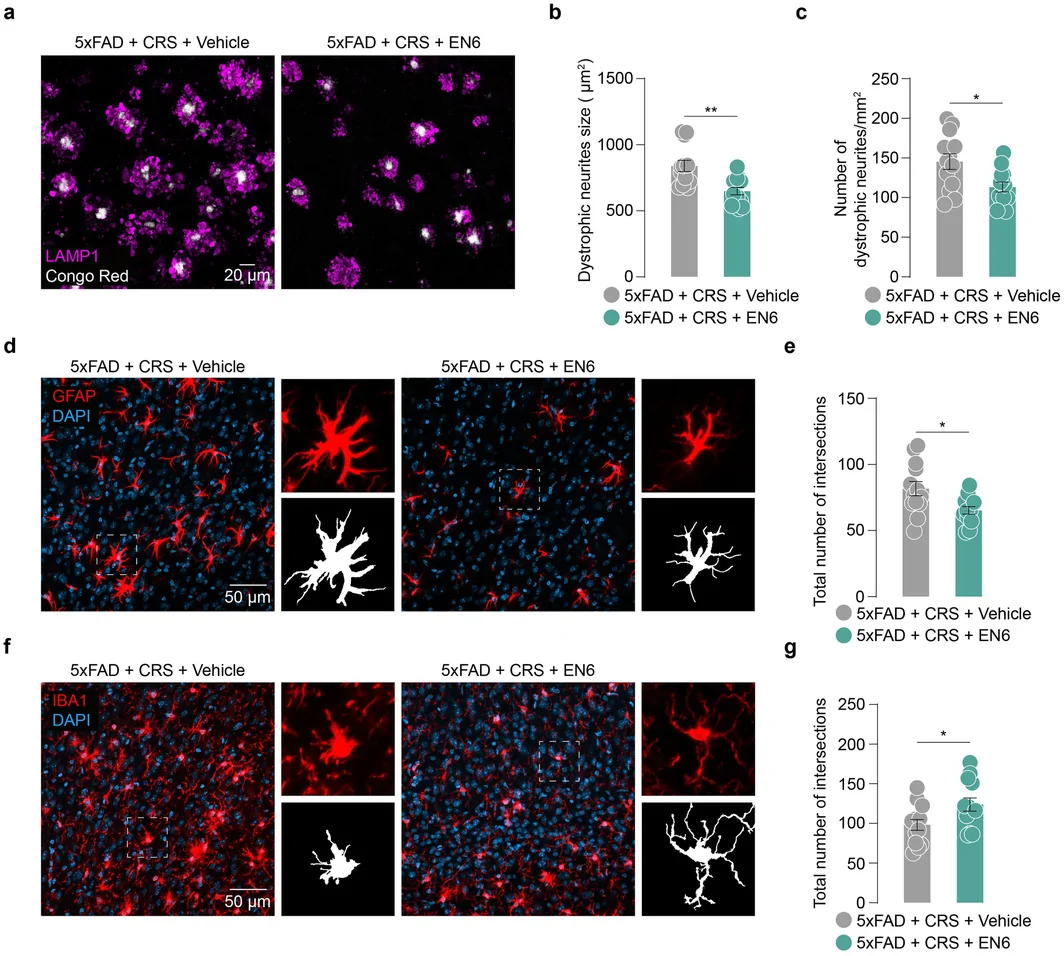
EN6 mitigates plaque‐associated axonal dystrophy and alleviates neuroinflammation in the depression‐AD comorbidity model. (a) Representative high‐resolution confocal micrographs demonstrating plaque‐associated dystrophic neurites in the PFC of 5xFAD + CRS + Vehicle and 5xFAD + CRS + EN6 group. (b) Statistical morphometric quantification of axonal pathology (*n* = 13 mice per group, 4–10 dystrophic neurites analyzed and averaged per mouse, Mann–Whitney test). (c) Statistical plaque‐associated dystrophic neurite density (*n* = 13 mice per group, 2–4 fields of view analyzed and averaged per mouse; Mann–Whitney test). (d) Confocal visualization of reactive astrogliosis in PFC. Left panels show low‐magnification overviews stained for GFAP and counterstained with DAPI. Right panels provide high‐magnification snapshots of individual isolated astrocytes alongside their corresponding binarized skeletonized masks used for morphological trace reconstructions. (e) Sholl analysis quantification displaying the total number of intersections per astrocyte (*n* = 13 mice per group, 3–4 astrocytes analyzed and averaged per mouse, Mann–Whitney test). (f) Morphological profiling of microglial activation phenotypes. Panoramic and detailed cropped views display PFC immunostained for IBA1 (red, microglia) and DAPI. Accompanying panels show high‐power individual cell architecture and binarized morphological skeleton profiles. (g) Statistical quantification of Sholl analysis total intersections per microglial (*n* = 13 mice per group, 3–4 microglia analyzed and averaged per mouse, Mann–Whitney test). Each dot represents an individual animal. Error bars represent mean ± SEM. *p < 0.05, **p < 0.01, ***p < 0.001, ns = no significance.

We next profiled the reactive states of cortical astrocytes and microglia. GFAP immunostaining and skeletonized reconstructions mapped reactive astrogliosis (Figure 6d). Sholl analysis demonstrated that EN6 intervention significantly reduced the total number of process intersections, reversing the pathological astrocytic hypertrophy observed in vehicle controls (Figure 6e). Concurrently, IBA1 staining and morphological tracking mapped microglial reactivity (Figure 6f). While vehicle‐treated comorbid mice displayed a marked reduction in branching complexity due to reactive amoeboid transformation, EN6 treatment induced a robust increase in process intersections (Figure 6g), indicating re‐ramification toward a homeostatic surveillance state.

To resolve this glial remodeling with high fidelity, we conducted extended single‐cell stereological profiling (Figure S6). For GFAP astrocytes, EN6 significantly dampened stress‐induced proliferation, contracting overall cell density (Figure S6a), while correcting hypertrophic activation by significantly reducing both soma area (Figure S6b) and total process length (Figure S6c). Conversely, for IBA1 microglia, EN6 suppressed inflammatory proliferation, decreasing cell density (Figure S6d) and alleviating somatic swelling (Figure S6e). Crucially, this somatic condensation was accompanied by a highly significant elongation of total process length per cell (Figure S6f), quantitatively confirming that EN6 rescues microglia from a pro‐inflammatory state.

## 4 Discussion

This study provides functional evidence linking CRS to accelerated AD pathology, underscoring the therapeutic relevance of modulating the mTORC1–autophagy axis. Through multi‐scale functional and histopathological assessments, we demonstrated that CRS intensifies network desynchronization, amyloid accumulation, and neuroinflammatory responses. Biochemical analyses and longitudinal tracking revealed that mTORC1 hyperactivation and accompanying autophagy‐related abnormalities may contribute to this pathology. Importantly, pharmacological inhibition of mTORC1 via EN6 mitigated these pathological cascades in an autophagy‐dependent manner, leading to reduced amyloid burden, attenuated gliosis, and restored cortical network coordination.

To resolve the clinical ambiguity surrounding the depression‐AD association [3, 8], we simulated environmental stress in 5xFAD mice. Chronic stress typically worsens the cerebral milieu via sustained hypothalamic–pituitary–adrenal (HPA) axis dysfunction and neurodestructive cascades [29, 30]. In alignment, our functional imaging demonstrated that stress reorganizes cortical network architecture, inducing micro‐scale single‐neuron hyperactivity and a breakdown of macroscopic slow‐wave synchrony. Beyond circuit phenomenology, transcriptomic and biochemical profiling unmasked that anomalous mTOR hyperactivation couples chronic stress to AD aggravation. This stress‐induced mTOR over‐activation operates as a molecular brake that impaired autophagic activity, establishing a permissive biochemical milieu for multi‐fraction Aβ accumulation. While our data demonstrate that stress‐induced mTORC1 hyperactivation impaired autophagic activity to elevate soluble Aβ accumulation and disrupt neural network coordination, existing literature establishes that elevated Aβ levels can, in turn, directly inhibit autophagic activity [31]. This reciprocal interaction supports a potential feed‐forward pathological loop: chronic stress initiates mTORC1‐driven autophagic failure and Aβ accumulation, while the resulting Aβ buildup further exacerbates autophagic suppression, thereby perpetuating network desynchronization and disease progression.

Depression research reveals that central autophagic machinery transitions from an adaptive “alarm/resistance” phase, where autophagic flux is dynamically recruited “on‐demand” to clear synaptic congestion, to a terminal “exhaustion” phase [24]. This conceptual framework reconciles the operational duality of mTORC1, which is heavily dictated by stress duration, regional heterogeneity, and pharmacodynamic kinetics. While rapid‐acting antidepressants like ketamine elicit an acute, transient burst of mTORC1 activation to stimulate synaptic protein synthesis and restore spine density [32], environmental stress triggers a distinct biphasic trajectory: acute stress temporarily dampens mTORC1 to promote autophagic flux, whereas prolonged chronic stress precipitates persistent mTORC1 hyperactivation [24]. In hyperactive cortical networks, this sustained over‐activation drives “persistent autophagy‐related abnormalities” that fuel proteotoxic Aβ accumulation and macro‐circuit desynchronization. Reconciling these temporal and regional divergences highlights that precise microenvironmental tuning defines the therapeutic window for targeting the mTORC1–autophagy axis in comorbid neurodegeneration.

The spatial–temporal interdependence between autophagic flux and Aβ biogenesis represents a critical nexus in AD [33, 34]. Synthesized APP and its primary cleavage enzyme, BACE1, converge and undergo amyloidogenic processing within the acidic microenvironment of endosomes [35, 36]. Under autophagic suppression, cargo degradation blockade precipitates pathological accumulation and swelling of endosomal compartments [37, 38]. This endosomal congestion prolongs the interaction time of APP and BACE1 within these sub‐cellular hubs, accelerating enzymatic cleavage and Aβ generation [39, 40, 41, 42]. Consistent with this framework, EN6 treatment was associated with reduced BACE1 abundance and decreased CTFβ generation, supporting reduced amyloidogenic APP processing. In contrast, ADAM10 and PS1 protein abundance remained unchanged. Because the enzymatic activities of BACE1, ADAM10, and γ‐secretase were not directly measured, these protein‐expression data should not be interpreted as direct evidence of altered secretase activity.

Crucially, stress‐associated autophagic impairment may contribute to network‐wide desynchronization. We propose that reducing cortical Aβ burden via EN6‐mediated autophagic rescue is intrinsically coupled to the mitigation of neuronal hyperactivity and restoration of SWA. Soluble Aβ oligomers impair synaptic glutamate reuptake, resulting in glutamate accumulation within the synaptic cleft that drives localized neuronal hyperactivity [15, 43, 44, 45]. This excitation/inhibition (E/I) disequilibrium fragments long‐range cortical synchronization and decouples distant brain regions, while plaque‐associated dystrophic neurites physically impede action potential propagation along compromised axons [15, 38, 44]. EN6 treatment lowered soluble Aβ levels and attenuated plaque pathology, alleviating the persistent synaptic toxicity that fuels over‐activation. This clearance sequentially dampens frontocortical circuit “noise,” allowing physiological SWA patterns to re‐emerge.

As a specific mTORC1 inhibitor [23], EN6 offers a distinct translational advantage by bridging sub‐cellular metabolic rejuvenation with macro‐scale network realignment. EN6 downregulates BACE1, reducing Aβ burden and rescuing network dynamics, diverging from conventional Aβ‐targeting monoclonal antibodies or non‐selective secretase inhibitors that carry risks of pan‐secretase cytotoxicity or neurovascular side effects.

Despite these multi‐scale insights, several limitations warrant cautious interpretation. First, while our multi‐scale neurofunctional imaging and transcriptomic profiling were geographically localized to the PFC, chronic stress‐induced neurodegeneration typically engages a broader, interconnected neural network. Notably, the hippocampus represents another vulnerable epicenter susceptible to stress‐driven pathology. Recent evidence demonstrates that CRS substantially elevates hippocampal IL‐17A levels, subsequently impairing PINK1/Parkin‐mediated mitophagy, reducing ATP production, and precipitating mitochondrial and synaptic ultrastructural deficits in the hippocampus [46]. This hippocampal mitochondrial energetic failure may act in concert with the cortical autophagic stagnation and circuit desynchronization identified in our study, together accelerating global systemic cognitive and emotional decline. Future network‐wide investigations incorporating multi‐brain region imaging techniques will be valuable to untangle the regional spatiotemporal crosstalk between the PFC and hippocampus. Second, although the coordinated changes in LC3B and p62, together with the chloroquine pathway‐dependency experiment, support altered autophagic activity, LC3B and p62 were not dynamically compared in the presence and absence of lysosomal inhibition. Therefore, these marker changes should not be interpreted as a direct quantitative measurement of autophagic flux. Third, our bulk tissue RNA‐seq masks cell‐type‐specific transcriptomic alterations within the heterogeneous cortical microenvironment. Given the highly divergent roles of autophagy across neurons [19], microglia [47], astrocytes [48], and oligodendrocytes [49], future studies employing single‐cell transcriptomics paired with cell‐type‐specific autophagic reporters are indispensable. Fourth, because the precise pharmacokinetic (PK) profiles and blood–brain barrier permeability of EN6 remain uncharacterized [23], this study utilized intracerebroventricular administration as a proof‐of‐concept intervention. While this localized delivery establishes the central therapeutic efficacy of target‐specific mTORC1 deactivation, translational clinical application will require extensive systemic formulation optimization and rigorous safety profiling.

## 5 Conclusion

In summary, our findings support a mechanistic framework in which chronic restraint stress accelerates AD‐related pathology through mTORC1 activation, autophagy‐related abnormalities, and subsequent Aβ accumulation. Furthermore, intervention with the mTORC1 inhibitor EN6 mitigates these pathological cascades, highlighting the mTORC1–autophagy axis as a potential therapeutic target for addressing stress‐aggravated neurodegenerative conditions.

## Author Contributions

Project design, Yang Zou; surgery and histology, Yang Zou, Jun Yang; behavioral experiments, Yang Zou, Yang Li; data interpretation and analysis, Yang Zou, Guodong Wang, Wen Lu; figure preparation, Yang Zou, Xiaoping Chen, Xingxing Ma, Kehan Chen; manuscript writing, Yang Zou, Peng Liang, Yinghui Wu, Tao Zhuo. All authors read and commented on the manuscript.

## Funding

This study is supported by the Guangxi Science and Technology Major Project (GuikeAA22096030), 2025 Clinical Rational Drug Use Practice Research (HXXT‐KY‐LCYY2025MS‐0145) and the 2025 Bethune Charitable Foundation Shining Across China‐Medicinal Research Fund (Z04J2025E170).

## Disclosure

Generative AI statement: The author(s) declared that generative AI was not used in the creation of this manuscript.

## Ethics Statement

All experimental procedures were strictly executed in compliance with the guidelines approved by the Institutional Animal Care and Use Committee of Guangxi Medical University (No. 202605001).

## Conflicts of Interest

The authors declare no conflicts of interest.

## Supporting information


**Table S1:** Detailed specifications of primary and secondary antibodies used for western blot and immunofluorescence profiling.
**Figure S1:** Detailed analysis of neuronal activity distribution reveals increased hyperactive and decreased normal neurons in the PFC of CRS‐exposed AD mice.
**Figure S2:** Extended profiling of cortical glial morphometric quantification under CRS.
**Figure S3:** Time‐course kinetics of mTORC1 hyperactivation, autophagic collapse, and Aβ accumulation during CRS exposure in 5xFAD mice.
**Figure S4:** Pharmacological mTOR activation by MHY1485 exacerbates autophagic impairment and soluble Aβ accumulation in 5xFAD mice.
**Figure S5:** EN6 inhibits mTORC1 and modulates autophagy‐related markers, while chloroquine attenuates its anti‐amyloidogenic effect.
**Figure S6:** Extended morphometric quantification of EN6‐mediated rescue on PFC glial morphology in comorbid 5xFAD + CRS mice.

## Data Availability

The data that support the findings of this study are available from the corresponding author upon reasonable request.
