# Supplementary material for: mTORC1 Activation Links Chronic Stress to Meso‐Circuit Desynchronization and Accelerated Amyloid Pathology via Autophagic Dysfunction
Source: CNS Neurosci Ther. 2026 Sep 28;32(10):e71174. doi: 10.1002/cns.71174 (PMC13618737; doi:10.1002/cns.71174)
Supplement: Supplementary file 1 [file CNS-32-e71174-s001.docx]

| Name | Company | Catalog number |
| --- | --- | --- |
| rat anti-LAMP1 | DSHB | 1D4B |
| rabbit anti-GFAP | Abcam | ab68428 |
| mouse anti-IBA1 | Abcam | ab283319 |
| donkey anti-rabbit Alexa Fluor 555 | Abcam | ab150062 |
| donkey anti-mouse Alexa Fluor 555 | Abcam | ab150110 |
| donkey anti-rat Alexa Fluor 647 | Abcam | ab150155 |
| rabbit anti-p-AMPK | CST | 2532 |
| rabbit anti-AMPK | CST | 2535 |
| rabbit anti-p-mTOR | Abcam | ab137133 |
| mouse anti-mTOR | Proteintech | 66888-1-Ig |
| rabbit anti-ACTB | BOSTER | BM3873 |
| rabbit anti-p-S6K | CST | 9234 |
| rabbit anti-S6K | CST | 34475 |
| rabbit anti-p-4EBP1 | CST | 2855 |
| rabbit anti-4EBP1 | CST | 9644 |
| rabbit anti-APP | CST | 2452 |
| mouse anti-6E10 | Biolegend | SIG-39300 |
| rabbit anti-ADAM10 | Abcam | ab124695 |
| rabbit anti-BACE1 | CST | 5606 |
| rabbit anti-Akt | CST | 9272 |
| rabbit anti-p-Akt | CST | 4060 |
| mouse anti-LC3B | CST | 83506 |
| rabbit anti-p62 | CST | 23214 |
| mouse anti-PS1 | Abcam | ab15458 |
| HRP-linked goat anti-mouse IgG | BOSTER | BA1050 |
| HRP-linked goat anti-rabbit IgG | BOSTER | BA1054 |

**Table S1 Detailed specifications of primary and secondary antibodies used for western blot and immunofluorescence profiling.**

**Figure S1 Detailed analysis of neuronal activity distribution reveals increased hyperactive and decreased normal neurons in the PFC of CRS-exposed AD mice.**

a-d. Fraction distribution histograms and corresponding pie charts illustrating the functional stratification of PFC neurons based on their spontaneous firing rates (calcium transients per minute) across four experimental cohorts: WT (n = 608 neurons), WT + CRS (n = 626 neurons), 5xFAD (n = 724 neurons), and 5xFAD + CRS (n = 670 neurons). Subpopulations are color-coded and classified into three distinct categories: silent neurons (0 transients/min, light blue), normal neurons (0 < frequency ≤ 5 transients/min, medium blue), and hyperactive neurons (frequency ≥ 6 transients/min, dark blue).

e-g. Statistical quantification comparing the fraction of total neurons per animal across groups for each neural subpopulation (n = 5 mice per group, one-way ANOVA).

h. Quantified summary of the overall mean calcium transient frequency per animal across the designated groups (n = 5 mice per group, one-way ANOVA).

Each dot represents an individual animal. Error bars represent mean ± SEM. *P < 0.05, **P < 0.01, ***P < 0.001, ns = no significance.

**Figure S2 Extended profiling of cortical glial morphometric quantification under CRS.**

a. Quantification of PFC astrocyte density (n = 12 mice per group, 3-4 fields of view analyzed and averaged per mouse, Mann-Whitney test).

b. Morphometric assessment of astrocytic soma area (n = 12 mice per group, 2-3 astrocytes analyzed and averaged per mouse, Mann-Whitney test).

c. Quantification of the total process length per astrocyte (n = 12 mice per group, 3-5 astrocytes analyzed and averaged per mouse, Mann-Whitney test).

d. Quantification of PFC microglial density (n = 12 mice per group, 3-5 fields of view analyzed and averaged per mouse, Mann-Whitney test).

e. Morphometric analysis of microglial soma area (n = 12 mice per group, 2-5 microglia analyzed and averaged per mouse, Mann-Whitney test).

f. Assessment of the total process length per microglial cell (n = 12 mice per group, 3-5 microglia analyzed and averaged per mouse, Mann-Whitney test).

Each dot represents an individual animal. Error bars represent mean ± SEM. *P < 0.05, **P < 0.01, ***P < 0.001, ns = no significance.

**Figure S3 Time-course kinetics of mTORC1 hyperactivation, autophagic collapse, and Aβ accumulation during CRS exposure in 5xFAD mice.**

a. Schematic diagram of the experimental paradigm for chronic restraint stress (CRS) exposure (0, 7, 14, and 30 days) in 5xFAD mice (n = 6 mice per group, one-way ANOVA).

b-g. Representative western blots and quantitative analysis of cortical (b, e) p-mTOR/mTOR, (c, f) p-S6K/S6K (mTORC1 substrate), and (d, g) p-AKT/AKT (mTORC2 substrate) levels at indicated CRS time points (n = 6 mice per group, one-way ANOVA).

h. Soluble Aβ42 concentrations in TBS-extracted cortical homogenates measured by ELISA across the CRS time course (n = 6 mice per group, one-way ANOVA).

i–k. Representative western blots (i) and quantitative analysis of (j) LC3B/ACTB and (k) p62/ACTB protein levels in the prefrontal cortex during CRS progression (n = 6 mice per group, one-way ANOVA).

Each dot represents an individual animal. Error bars represent mean ± SEM. *P < 0.05, **P < 0.01, ***P < 0.001, ns = no significance.

**Figure S4 Pharmacological mTOR activation by MHY1485 exacerbates autophagic impairment and soluble Aβ accumulation in 5xFAD mice.**

a. Experimental paradigm for vehicle (Veh) or MHY1485 administration in 5xFAD mice. MHY1485 (CAS 326914-06-1, MCE, HY-B0795) was dissolved in 5% DMSO and 95% artificial cerebrospinal fluid (ACSF). A target dose of 0.4 nmol/mouse was calculated based on the effective in vitro concentration (10 μM) and mouse CSF volume. Accordingly, 1 μl of 400 μM MHY1485 was unilaterally delivered via intracerebroventricular (i.c.v.) microinjection twice weekly for 4 weeks prior to sacrifice.

b–d. Representative western blots (b) and quantitative analysis of cortical (c) LC3B/ACTB and (d) p62/ACTB protein levels in vehicle- and MHY1485-treated 5xFAD mice (n = 6 mice per group, Mann-Whitney test).

e. Soluble Aβ42 concentrations in TBS-extracted cortical homogenates measured by ELISA following MHY1485 treatment (n = 6 mice per group, Mann-Whitney test).

Each dot represents an individual animal. Error bars represent mean ± SEM. *P < 0.05, **P < 0.01, ***P < 0.001, ns = no significance.

**Figure S5 EN6 inhibits mTORC1 and modulates autophagy-related markers, while chloroquine attenuates its anti-amyloidogenic effect.**

a-f. Representative western blots and quantitative analysis of cortical (a, b) p-mTOR/mTOR, (c, d) p-S6K/S6K (mTORC1 substrate), and (e, f) p-AKT/AKT (mTORC2 substrate) levels in 5xFAD + CRS mice treated with vehicle or EN6 (n = 6 mice per group, Mann-Whitney test).

g-i. Representative western blots (g) and quantitative analysis of (h) LC3B/ACTB and (i) p62/ACTB protein levels in EN6-treated 5xFAD + CRS mice (n = 6 mice per group, Mann-Whitney test).

j. Schematic diagram of the experimental paradigm for CRS, EN6, and chloroquine (CQ) co-treatment in 5xFAD mice. Chloroquine (CAS 54-05-7, MCE, HY-17589A) was dissolved in artificial cerebrospinal fluid (ACSF). A target dose of 2.0 nmol/mouse was calculated based on the effective in vitro concentration (50 μM) and mouse CSF volume. Accordingly, 1 μl of 2000 μM chloroquine was unilaterally delivered via intracerebroventricular (i.c.v.) microinjection twice weekly for 4 weeks prior to sacrifice.

k. Soluble Aβ42 concentrations in TBS-extracted cortical homogenates measured by ELISA following EN6 alone or EN6 + CQ treatment (n = 6 mice per group, Mann-Whitney test).

Each dot represents an individual animal. Error bars represent mean ± SEM. *P < 0.05, **P < 0.01, ***P < 0.001, ns = no significance.

**Figure S6 Extended morphometric quantification of EN6-mediated rescue on PFC glial morphology in comorbid 5xFAD+CRS mice.**

a. Quantification of PFC astrocyte density (n = 13 mice per group, 2-3 fields of view analyzed and averaged per mouse, Mann-Whitney test).

b. Morphometric assessment of astrocytic soma area (n = 13 mice per group, 3-4 astrocytes analyzed and averaged per mouse, Mann-Whitney test).

c. Quantification of the total process length per astrocyte (n = 13 mice per group, 3-4 astrocytes analyzed and averaged per mouse, Mann-Whitney test).

d. Quantification of PFC microglial density (n = 13 mice per group, 3 fields of view analyzed and averaged per mouse, Mann-Whitney test).

e. Morphometric analysis of microglial soma area (n = 13 mice per group, 3-5 microglia analyzed and averaged per mouse, Mann-Whitney test).

f. Assessment of the total process length per microglial cell (n = 13 mice per group, 3-4 microglia analyzed and averaged per mouse, Mann-Whitney test).

Each dot represents an individual animal. Error bars represent mean ± SEM. *P < 0.05, **P < 0.01, ***P < 0.001, ns = no significance.
